# Supplementary material for: Seminoma and Embryonal Carcinoma Footprints Identified by Analysis of Integrated Genome-Wide Epigenetic and Expression Profiles of Germ Cell Cancer Cell Lines
Source: PLoS One. 2014 Jun 2;9(6):e98330. doi: 10.1371/journal.pone.0098330 (PMC4041891; doi:10.1371/journal.pone.0098330)
Supplement: File S1 — ZIP file containing DMRforPairs output for significant regions. Please start from the html files. (ZIP) [file pone.0098330.s008.zip › figures/39974.pdf]

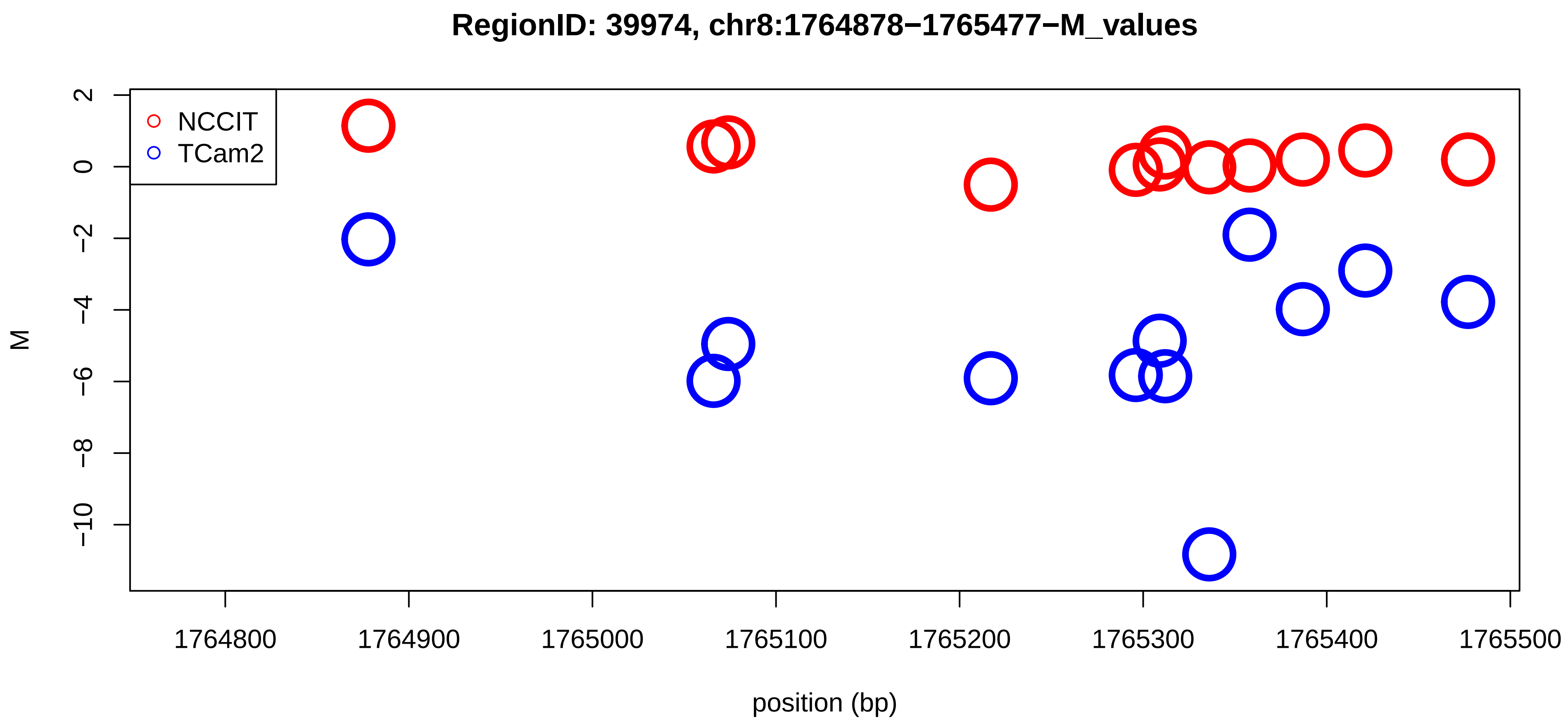
RegionID: 39974, chr8:1764878−1765477−M_values
NCCIT
TCam2
M
2
0
−2
−4
−6
−8
−10
1764800
1764900
1765000
1765100
1765200
1765300
1765400
1765500
position (bp)

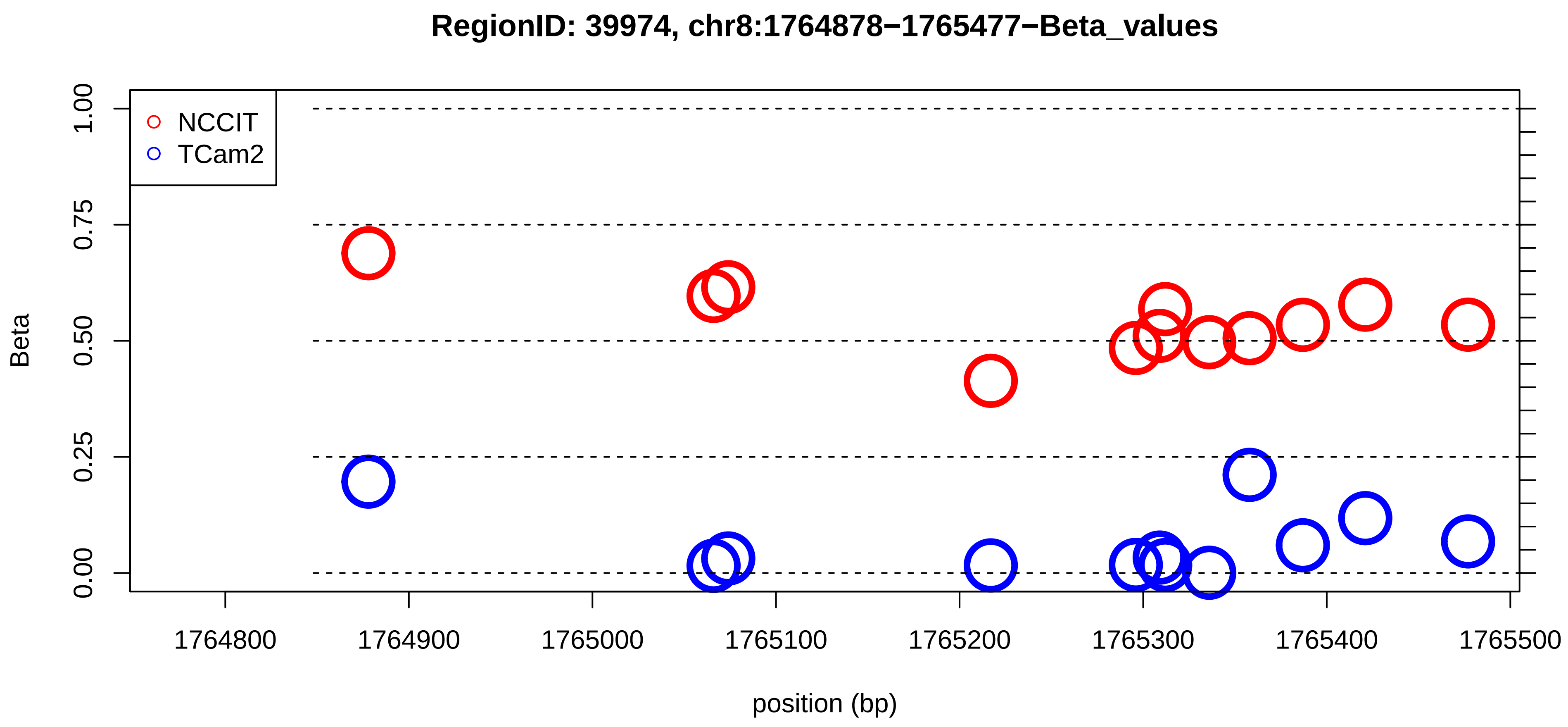
RegionID: 39974, chr8:1764878−1765477−Beta_values
NCCIT
TCam2
Beta
1.00
0.75
0.50
0.25
0.00
1764800
1764900
1765000
1765100
1765200
1765300
1765400
1765500
position (bp)

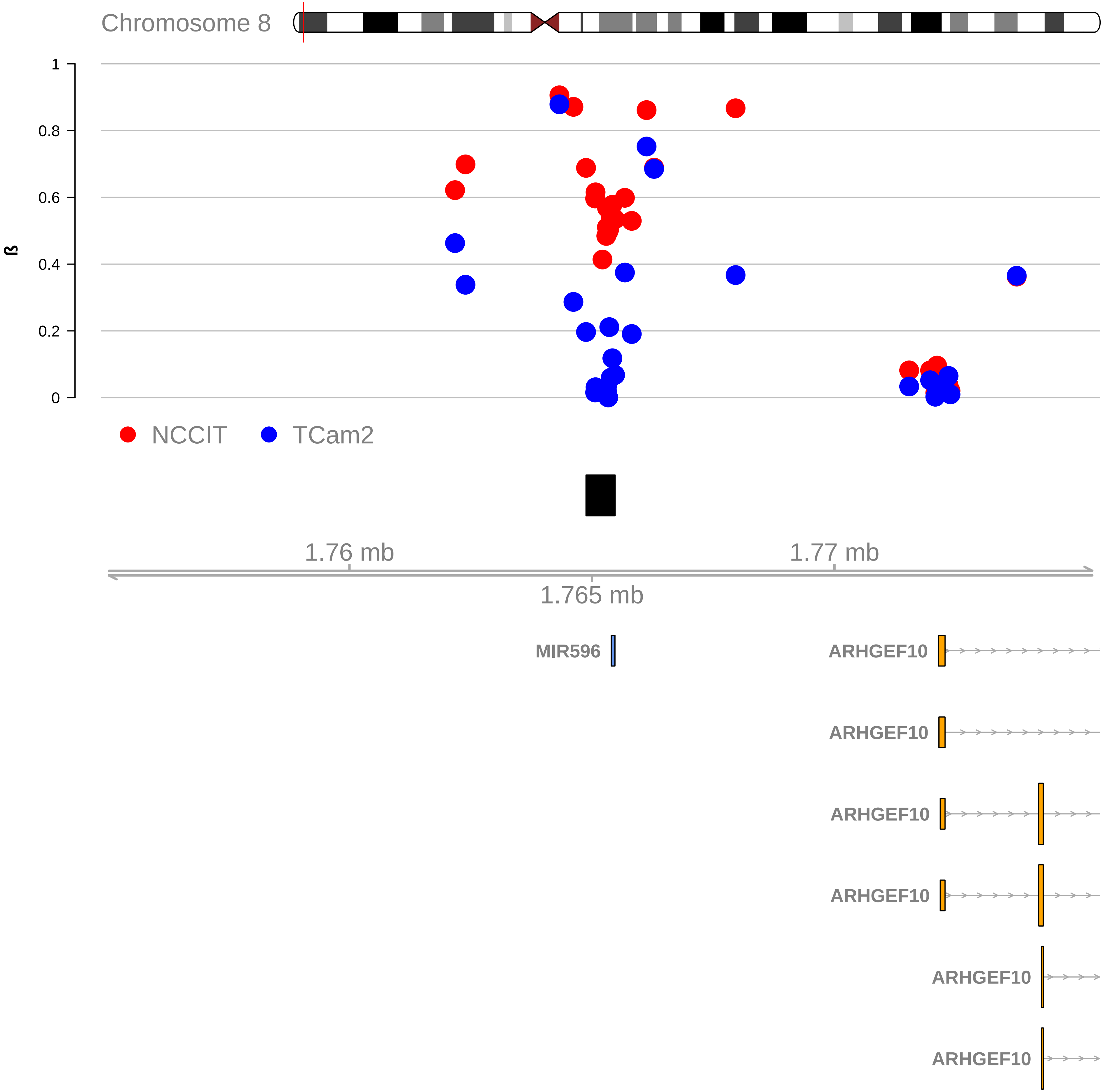

Chromosome 8
1
0.8
0.6
0.4
0.2
0
ß
NCCIT
TCam2
1.76 mb
1.765 mb
1.77 mb
MIR596
ARHGEF10
ARHGEF10
ARHGEF10
ARHGEF10
ARHGEF10
ARHGEF10
